# Supplementary material for: Mechanism of synergistic activation of Arp2/3 complex by cortactin and WASP-family proteins
Source: Nat Commun. 2023 Oct 28;14:6894. doi: 10.1038/s41467-023-42229-y (PMC10613254; doi:10.1038/s41467-023-42229-y)
Supplement: Supplementary file 1 — Supplementary Information [file 41467_2023_42229_MOESM1_ESM.pdf]

Supplementary Information for

Mechanism of Synergistic Activation of Arp2/3 complex by Cortactin and WASP-Family Proteins

Fred E. Fregoso, Malgorzata Boczkowska, Grzegorz Rebowski, Peter J. Carman, Trevor van Eeuwen, and Roberto Dominguez

Correspondence: Roberto Dominguez
Email: droberto@pennmedicine.upenn.edu

This file includes:

Supplementary Fig. 1	Cryo-EM data processing and workflow
Supplementary Fig. 2	Map validation
Supplementary Fig. 3	Western blot analysis of crosslinking reactions
Supplementary Fig. 4	Cortactin constructs used in this study
Supplementary Fig. 5	Cosedimentation of cortactin constructs with F-actin
Supplementary Fig. 6	Interaction of $Cort_{1-401}$ with F-actin
Supplementary Fig. 7	Binding of Arp2/3 complex to F-actin with or without cortactin
Supplementary Fig. 8	Alignment of the Arp2/3 complex and F-actin binding regions of cortactin
Supplementary Table 1	Primers and antibodies used in this study

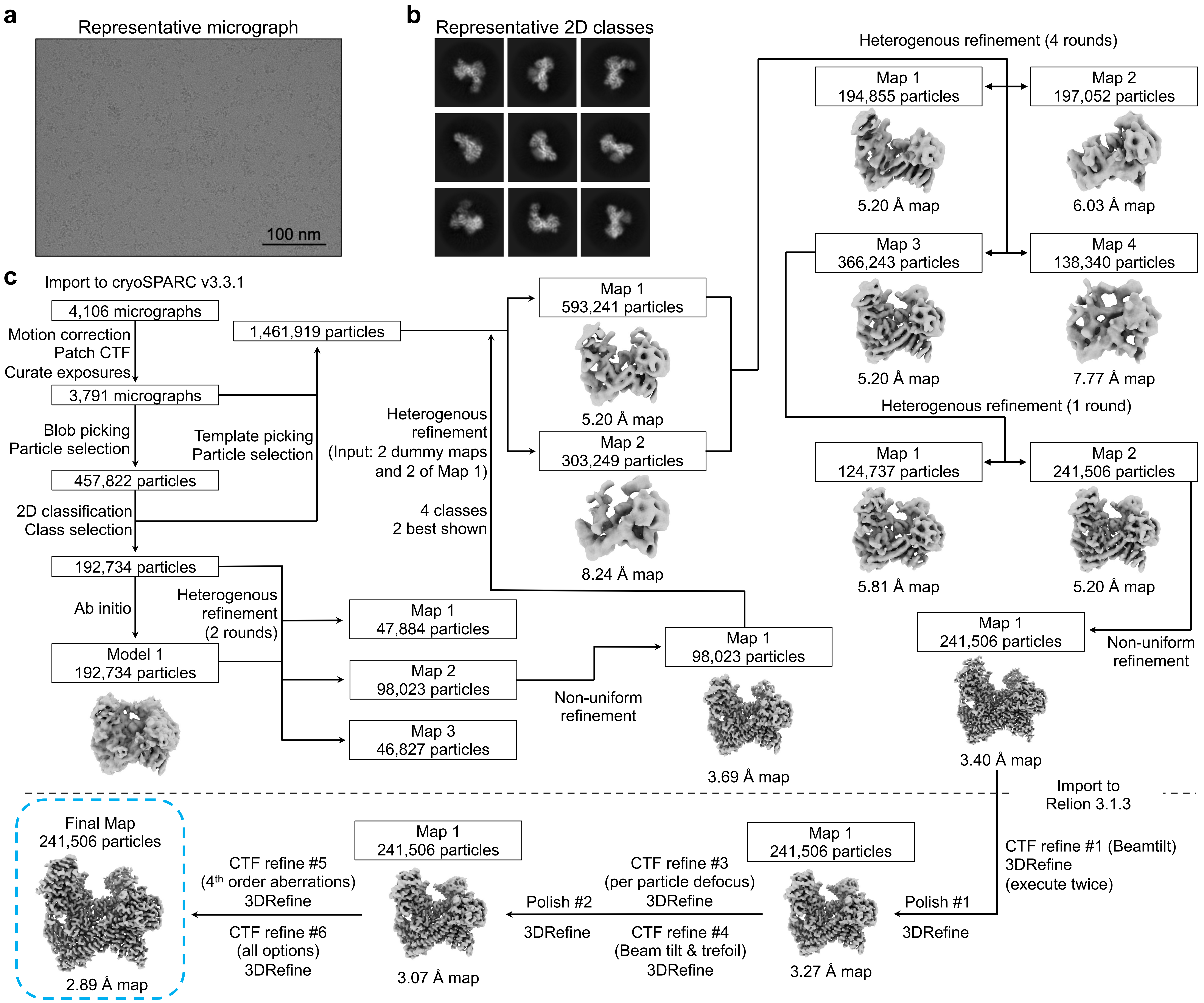

Supplementary Fig. 1: Cryo-EM data processing and workflow. a Representative micrograph of Cort$_{1\text{-}76}$-bound Arp2/3 complex. **b** cryoSPARC-generated 2D class averages. **c** Cryo-EM workflow (see Methods). A total of 4,106 micrographs were used to blob pick 457,822 particles. A subset of 192,734 particles were used to generate the first *ab initio* model. Two rounds of heterogenous classification using this model as reference yielded a map with 98,023 particles that revealed orphan density on Arp3 that was attributed to Cort$_{1\text{-}76}$. Non-uniform refinement then yielded a 3.69 Å reconstruction used as reference for subsequent rounds of heterogenous refinement. A total of 1,461,919 particles picked using selected 2D classes as template were used for heterogenous refinement with the reference map as input. This yielded four classes, two of which were taken through four iterative rounds of heterogenous refinement, resulting in a best class (366,243 particles) with good particle distribution and map quality. One round of heterogenous refinement and non-uniform refinement of this class yielded a 3.40 Å map comprising 241,506 particles. These particles were then transferred to Relion for iterative rounds of CTF and 3D refinement, producing a final map at 2.89 Å resolution after Bayesian polishing. Post processing of this map was performed with the program deepEMhancer.

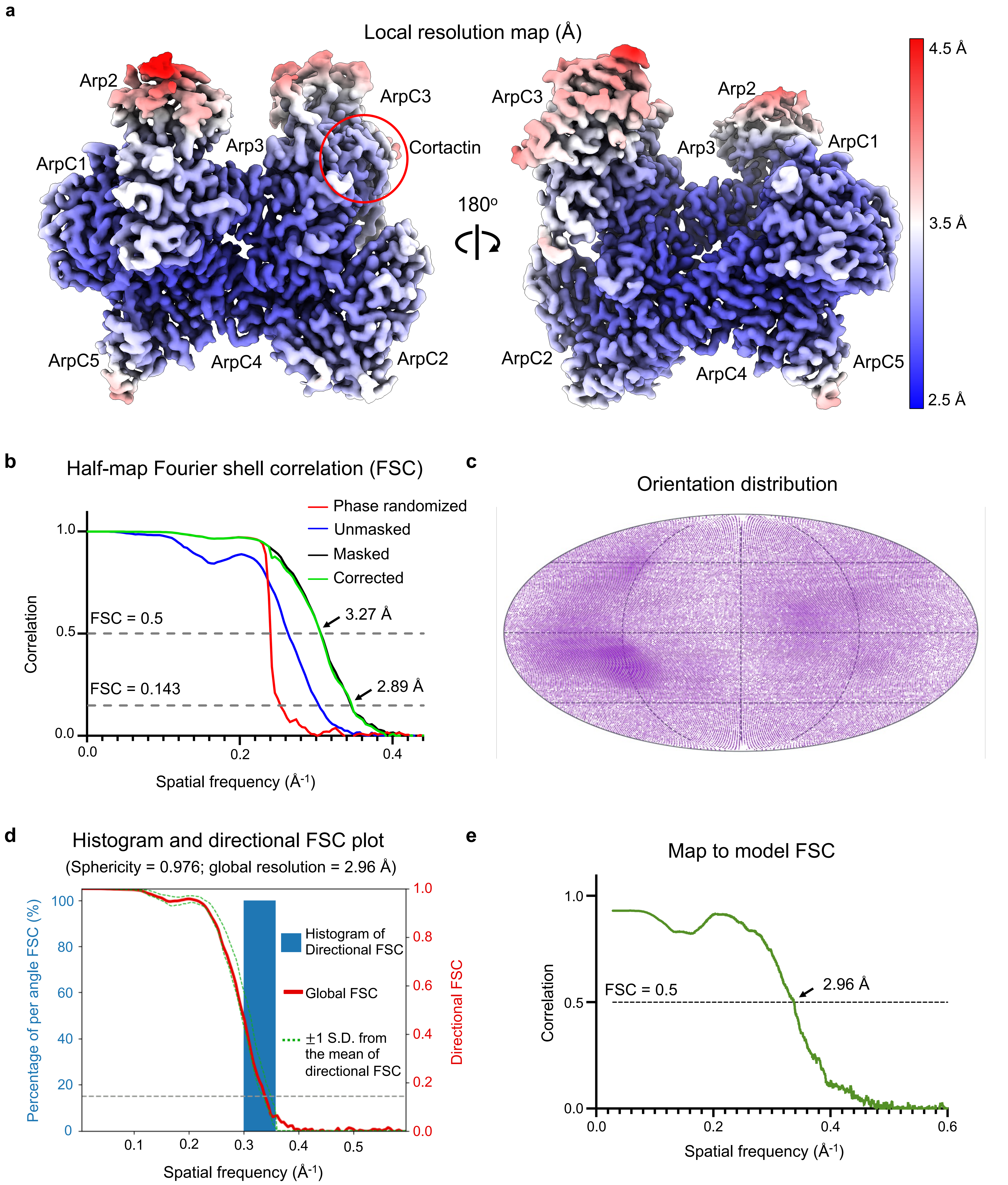

Supplementary Fig. 2: Map validation. a Cryo-EM map of Cort$_{1\text{-}76}$-bound Arp2/3 complex colored by resolution (as indicated by a side bar). Labels indicate the locations of Arp2/3 complex subunits. **b** The resolution of the map is 2.89 Å at FSC = 0.143. **c** Orientation distribution of particles from the final reconstruction as determined with the program cryoEF. The final map has a calculated efficiency (E_{od}) of 0.71. **d** 3D Fourier shell correlation calculated using the 3D-FSC server. The map shows high sphericity (0.976 out of 1) and a global resolution of 2.96 Å. **e** Map to model FSC calculated with the program Phenix.

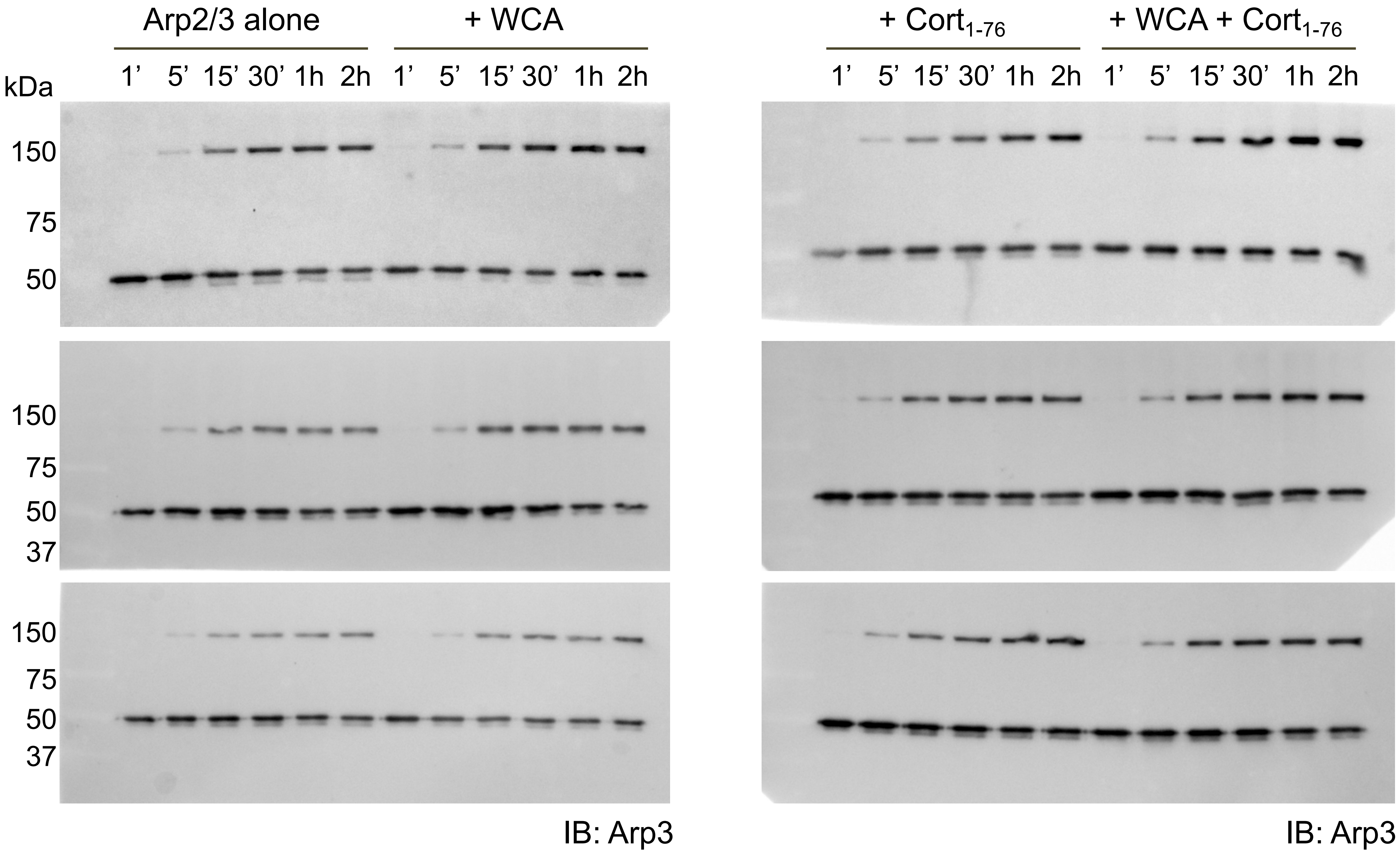

Supplementary Fig. 3: Western blot analysis of crosslinking reactions. Western blots (Arp3 antibody, 1:5000 dilution, Santa Cruz Biotechnology, sc-48344) used in quantifications of crosslinking reactions of Arp2/3 complex alone and with WCA, $Cort_{1\text{-}76}$, or WCA + $Cort_{1\text{-}76}$. The densitometric quantifications are shown in Fig. 2, and the source data are provided in the Source Data file (Figure 2 sheet). For each timepoint, quantifications were done by dividing the intensity of the crosslinked band (top) by the total Arp3 signal (top + bottom bands).

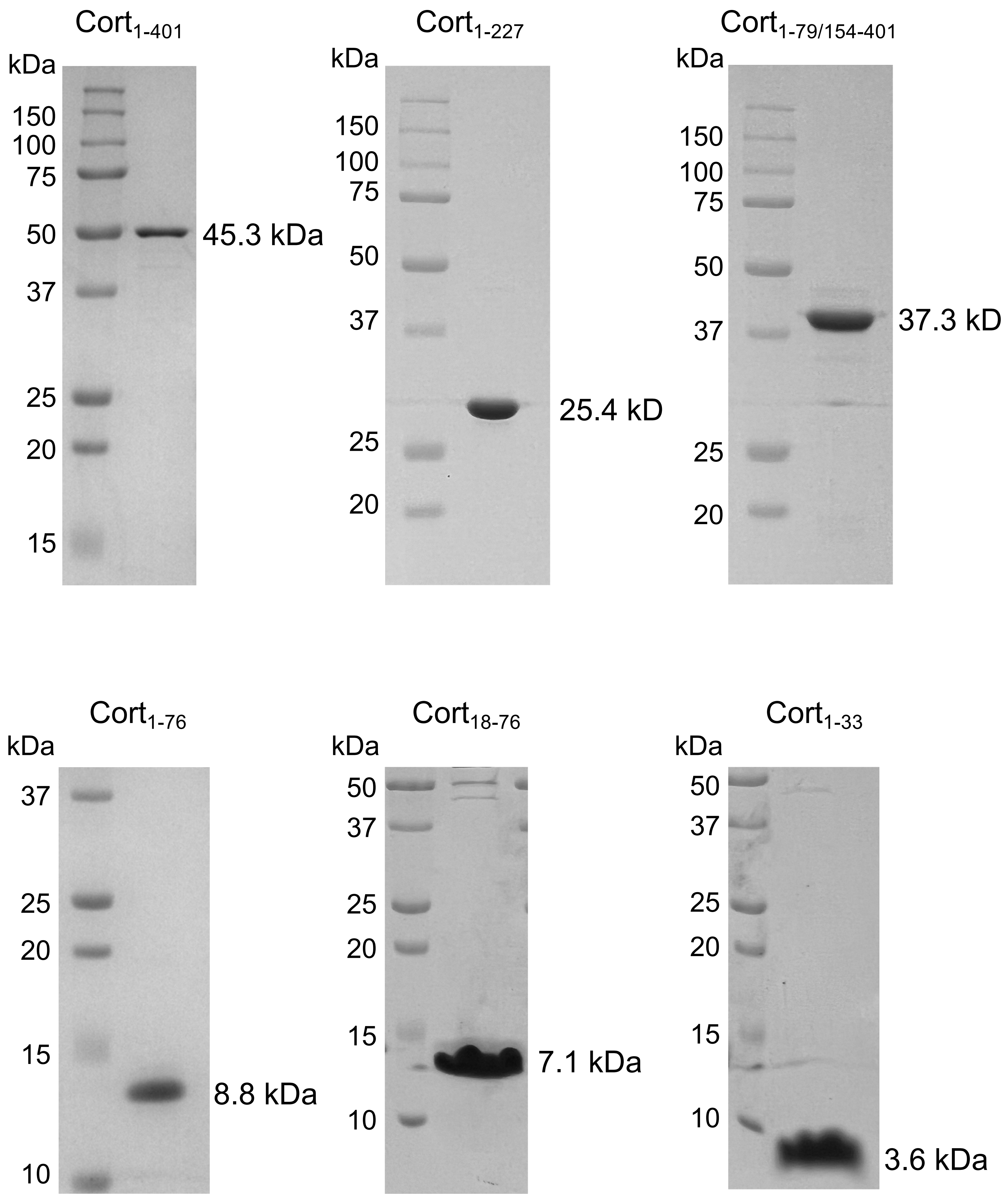

Supplementary Fig. 4: Cortactin constructs used in this study. Coomassie stained SDS-PAGE characterization of cortactin constructs used in this study.

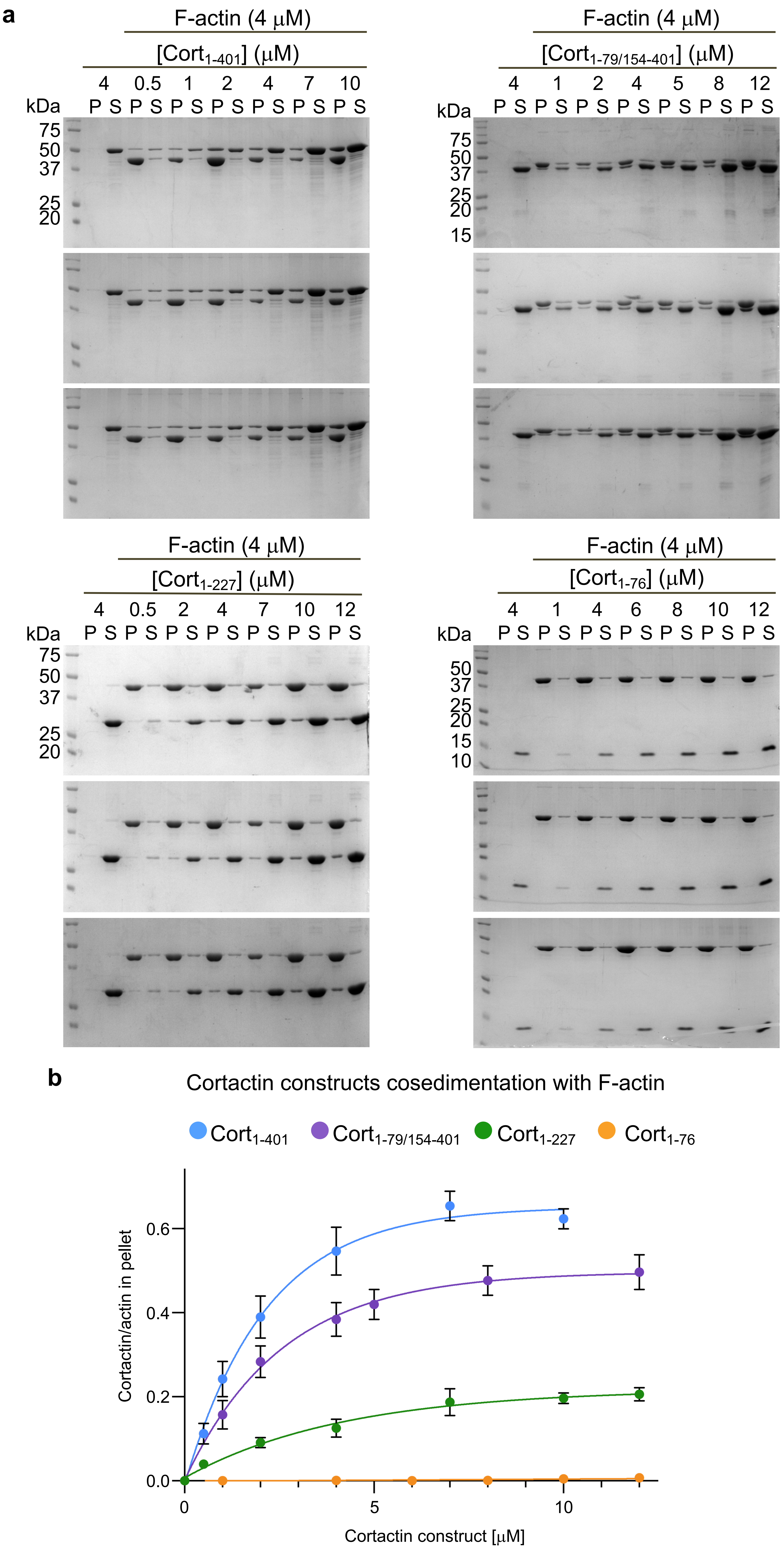

Supplementary Fig. 5: Cosedimentation of cortactin constructs with F-actin. a Coomassie stained SDS-PAGE of F-actin (4 μM) cosedimentations with cortactin constructs at the indicated concentrations (P and S, pellet and supernatant fractions, respectively). A schematic description of the cortactin constructs is shown in Fig. 3a. In all the gels, the actin band (43 kDa) appears between the 37 and 50 kDa molecular weight markers. **b** Densitometric quantification of the cosedimentation experiments shown in part ***a*** (n = 3). For each condition, quantifications were done by dividing the intensity of the band of the cortactin construct by that of actin in the pellet fraction. Data were fit to a first-order exponential equation and are reported as mean value ± standard deviation. The source data are provided as a Source Data file (S. Figure 5 sheet).

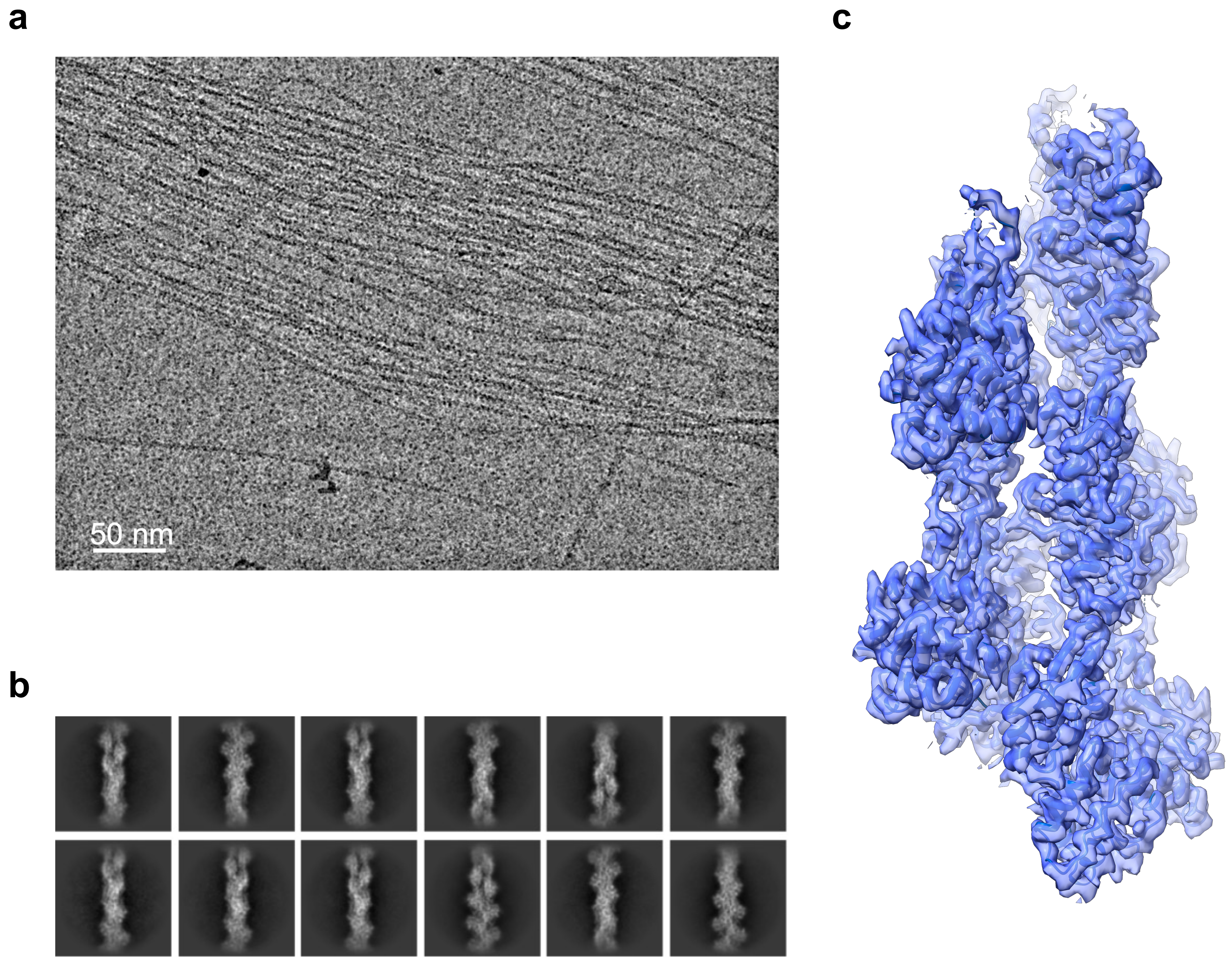

F-actin : $Cort_{1-401}$	
Magnification	64,000x
Voltage, keV	300
Total electron exposure, e/Å^2	50
Defocus range, mm	-0.8 – -2.5 μm
Pixel size, Å	0.68
Symmetry	C1
Initial number of particles	280,243
Final number of particles	264,652
Map resolution, Å	
FSC threshold 0.143 (0.5)	2.77 (3.08)
Resolution range	2.7 – 4.1

Supplementary Fig. 6: Interaction of $Cort_{1-401}$ with F-actin. a Cryo-EM micrograph of F-actin bundles formed by $Cort_{1-401}$. Scale bar 50 nm. **b** cryoSPARC-generated 2D class averages. **c** Cryo-EM map at 2.77 Å resolution obtained from helical reconstruction of actin filaments in the bundles formed by $Cort_{1-401}$. The map (transparent surface representation) was fit with an F-actin model (ribbon representation, PDB code 8F8P), showing no extra density corresponding to $Cort_{1-401}$. Data collection parameters are shown on the right.

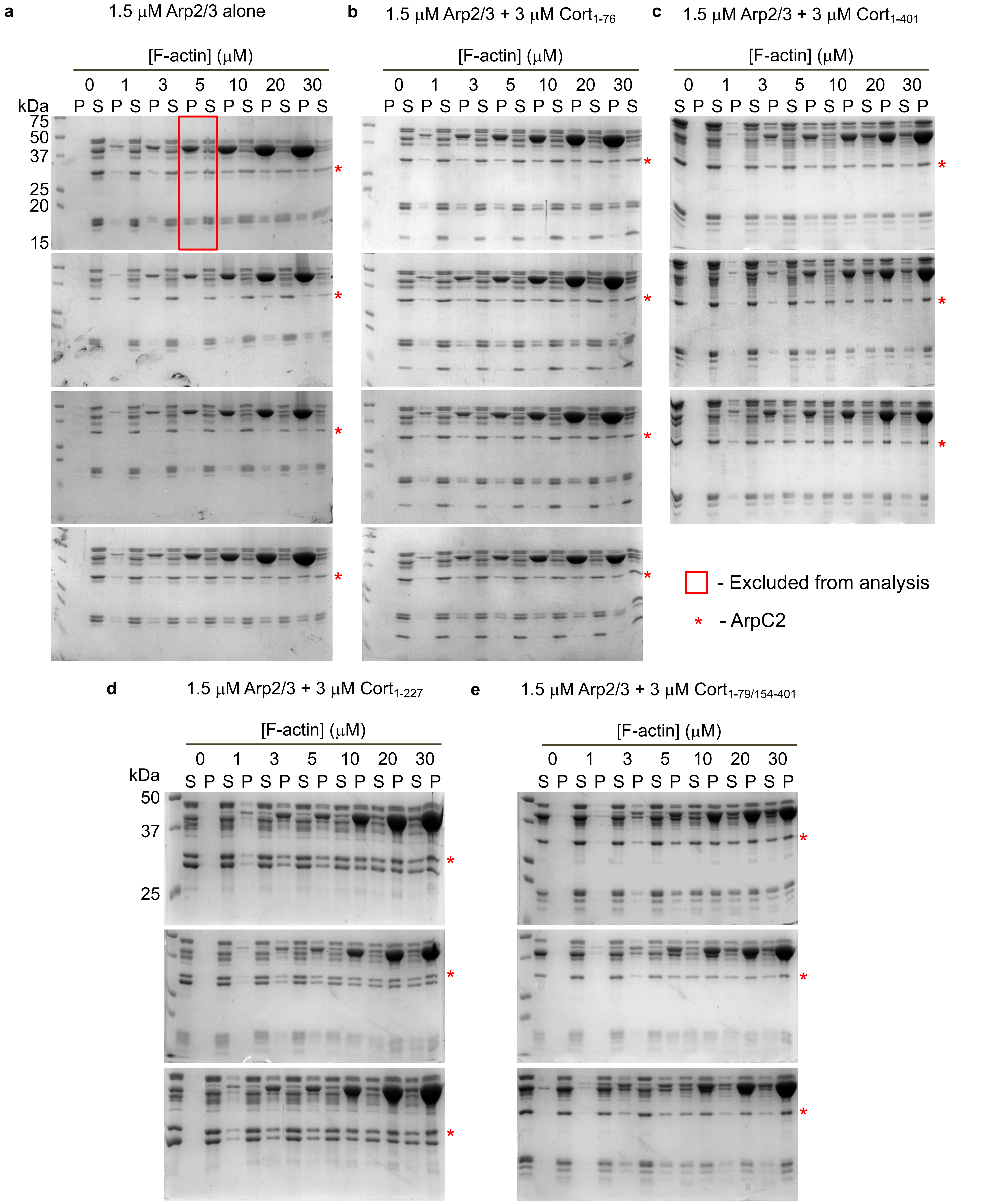

Supplementary Fig. 7: Binding of Arp2/3 complex to F-actin with and without cortactin constructs. a-e SDS-PAGE analysis of cosedimentation of 1.5 μM Arp2/3 complex with F-actin (at the indicated concentrations) in the absence or the presence of 3 μM cortactin constructs $Cort_{1\text{-}76}$, $Cort_{1\text{-}401}$, $Cort_{1\text{-}227}$, and $Cort_{1\text{-}79/154\text{-}401}$ (P and S, pellet and supernatant fractions, respectively). The densitometric quantification is based on subunit ArpC2, which is the most clearly defined in all the gels (indicated by a red asterisk). For each condition, quantifications were done by dividing the intensity of the band of ArpC2 in the pellet fraction by the total intensity of the bands of ArpC2 in the pellet and supernatant fractions. A red rectangle indicates a condition that could not be quantified and was excluded from analysis. The densitometric quantifications are shown in Fig. 3a, and the source data are provided in the Source Data file (Figure 3a sheet).

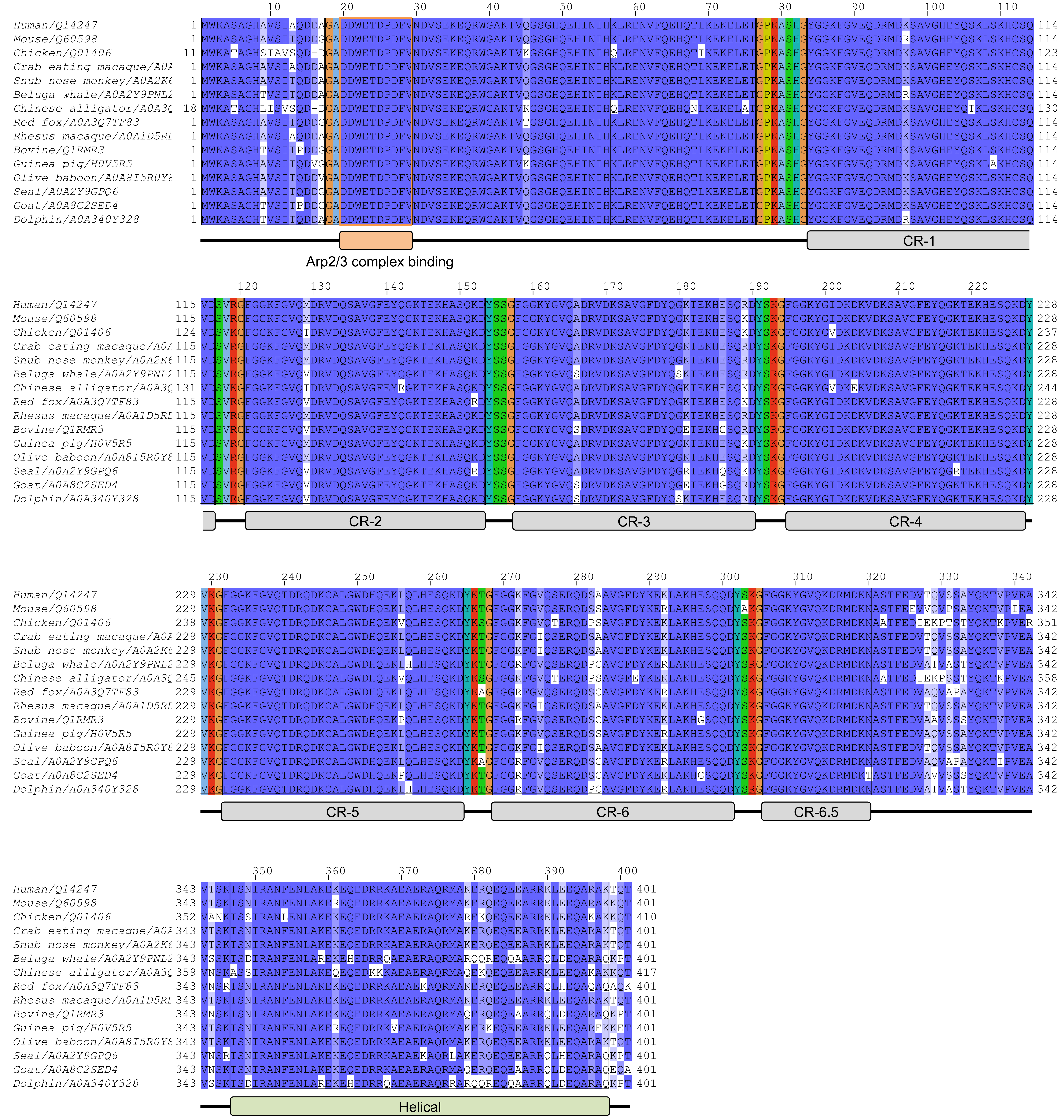

Supplementary Fig. 8: Alignment of the Arp2/3 complex and F-actin binding regions of cortactin. Sequence alignment of the Arp2/3 complex- and F-actin-binding regions of cortactin from different species. UniProt accession codes and the source are listed with the name of each sequence. A domain diagram indicates the location of the Arp2/3 complex-binding motif (orange), the cortactin repeats (CRs, gray) and the F-actin-binding Helical region (green). Coloring is according to conservation, from high (dark blue) to low (white). The linker regions are colored by amino acid type.

Supplementary Table 1. Primers and antibodies used in this study

Primer name	Primer sequence	Purpose
Cortactin_for_BamH1	GGAGGATCCATGTGGAAAGCCTCTGCAGGCCATGC	Cloning $Cort_{1-401}$
Cortactin_rev_33_EcoR1	TTCGAATTCCTAACTCACATCATTCACAAAATCAGGATC	Cloning $Cort_{1-33}$
Cortactin_rev_77_EcoR1	TTCGAATTCCTACGTTTCCAGCTCCTTCTCCTTG	Cloning $Cort_{1-76}$
Cortactin_rev_cr4_EcoR1	TTCGAATTCCTAGTCTTTCTGGGATTCGTGCTTCTCTG	Cloning $Cort_{1-227}$
Cortactin_rev_401_EcoR1	TTCGAATTCCTACGTCTGCTTCTTGGCTCTGGC	Cloning $Cort_{1-401}$
Cortactin_rev_Helix/CR1	CTTGGGTCCCGTTTCCAGCTCCTTC	Cloning $Cort_{1-79/154-401}$
Cortactin_for_Helix/CR3	GGACCCAAGTACTCTAGTGGCTTCGGTGGC	Cloning $Cort_{1-79/154-401}$
Cortactin_for_G18	TTCGGATCCGGAGCTGATGACTGGGAGACTGATCC	Cloning $Cort_{18-76}$

Antibody	Dilution	Description	Source	Cat #
**Primary antibodies**				
Arp3	1:5000	Mouse monoclonal anti-arp3 Ig	Santa Cruz Biotechnology	sc-48344
**Secondary antibodies**				
Anti-mouse IgG-HRP	1:10000	Anti-mouse HRP-linked Ig	Cell Signaling Technology	7076S
