# Supplementary material for: Mechanism of synergistic activation of Arp2/3 complex by cortactin and WASP-family proteins
Source: Nat Commun. 2023 Oct 28;14:6894. doi: 10.1038/s41467-023-42229-y (PMC10613254; doi:10.1038/s41467-023-42229-y)
Supplement: Supplementary file 3 — Description of Additional Supplementary Files [file 41467_2023_42229_MOESM3_ESM.pdf]

Description of Additional Supplementary Files

File Name: Supplementary Movie 1

Description: Full rotation of the cryo-EM map of $Cort_{1\text{-}76}$ (orange) bound to Arp2/3 complex. Arp2/3 complex subunits are colored as follows: Arp2, cyan; Arp3, green; ArpC1, yellow; ArpC2, ArpC3, and ArpC5, light gray; ArpC4, dark gray (see also **Fig. 1b** in the main text).
