# Supplementary material for: Mechanism of synergistic activation of Arp2/3 complex by cortactin and WASP-family proteins
Source: Nat Commun. 2023 Oct 28;14:6894. doi: 10.1038/s41467-023-42229-y (PMC10613254; doi:10.1038/s41467-023-42229-y)
Supplement: Supplementary file 5 — Reporting Summary [file 41467_2023_42229_MOESM5_ESM.pdf]

nature portfolio

Corresponding author(s): Roberto Dominguez

Last updated by author(s): Sep 20, 2023

Reporting Summary

Nature Portfolio wishes to improve the reproducibility of the work that we publish. This form provides structure for consistency and transparency in reporting. For further information on Nature Portfolio policies, see our Editorial Policies and the Editorial Policy Checklist.

Statistics

For all statistical analyses, confirm that the following items are present in the figure legend, table legend, main text, or Methods section.

n/a	Confirmed	
☐	☒	The exact sample size (*n*) for each experimental group/condition, given as a discrete number and unit of measurement
☐	☒	A statement on whether measurements were taken from distinct samples or whether the same sample was measured repeatedly
☐	☒	The statistical test(s) used AND whether they are one- or two-sided<br>*Only common tests should be described solely by name; describe more complex techniques in the Methods section.*
☒	☐	A description of all covariates tested
☒	☐	A description of any assumptions or corrections, such as tests of normality and adjustment for multiple comparisons
☐	☒	A full description of the statistical parameters including central tendency (e.g. means) or other basic estimates (e.g. regression coefficient) AND variation (e.g. standard deviation) or associated estimates of uncertainty (e.g. confidence intervals)
☐	☒	For null hypothesis testing, the test statistic (e.g. *F*, *t*, *r*) with confidence intervals, effect sizes, degrees of freedom and *P* value noted<br>*Give P values as exact values whenever suitable.*
☒	☐	For Bayesian analysis, information on the choice of priors and Markov chain Monte Carlo settings
☒	☐	For hierarchical and complex designs, identification of the appropriate level for tests and full reporting of outcomes
☒	☐	Estimates of effect sizes (e.g. Cohen's *d*, Pearson's *r*), indicating how they were calculated

Our web collection on statistics for biologists contains articles on many of the points above.

Software and code

Policy information about availability of computer code

Data collection	EPU software (Thermofisher) was used for cryo-EM data collection.
Data analysis	Data processing for cryo-EM and single particle anaylsis, MotionCorr2, Relion-3.0.8, CTFFind-4.1.13, and cryoSPARC-3.3.1. Post processing of the Cort1-76-Arp2/3 complex map was performed using deepEMhancer, and the orientation distribution of particles from the final reconstruction was determined using cryoEF-v1.1.0. The 3DFSC server was used to calculate the 3D-FSC of the final map. The program Coot-0.9.3 was used for model building, and the program Phenix-1.2.1-4487 was used for model refinement. Map figures were prepared using the programs ChimeraX-1.6.1 and PyMOL-2.5.2. For ITC analyses, the program Origin-7.0 was used to analyze the raw binding isotherms. Domain diagrams were made in PowerPoint-16.53, multiple sequence alignment was performed in Jalview-2.11.1.4 using ClustalW-1.2.2. For pyrene-actin polymerization and crosslinking assay, Prism-9.0 was used for statistical analysis and graphing. For cosedimentation experiments, the program ImageLab-6.0.1 build 34 was used for densitometric analysis.

For manuscripts utilizing custom algorithms or software that are central to the research but not yet described in published literature, software must be made available to editors and reviewers. We strongly encourage code deposition in a community repository (e.g. GitHub). See the Nature Portfolio guidelines for submitting code & software for further information.

Data

Policy information about availability of data

All manuscripts must include a data availability statement. This statement should provide the following information, where applicable:

- Accession codes, unique identifiers, or web links for publicly available datasets
- A description of any restrictions on data availability
- For clinical datasets or third party data, please ensure that the statement adheres to our policy

Data availability

The cryo-EM map and atomic model generated for this work is deposited in the Electron Microscopy Data Bank and Protein Data Bank with accession codes EMD-41135 [https://www.ebi.ac.uk/emdb/EMD-41135] and 8TAH [https://doi.org/10.2210/pdb8tah/pdb], respectively. The PDB accession codes for existing models referenced here are 6UHC [https://doi.org/10.2210/pdb6UHC/pdb] and 7JPN [https://doi.org/10.2210/pdb7JPN/pdb]. Uniprot database was used to obtain the sequence for mouse cortactin Q60598 [https://www.uniprot.org/uniprotkb/Q60598/entry]. Uncropped western blots and gels used in quantifications for this study are shown in Supplementary Information. The biochemical data generated in this study are provided in the Source Data file. Source data are provided with this paper.

Research involving human participants, their data, or biological material

Policy information about studies with human participants or human data. See also policy information about sex, gender (identity/presentation), and sexual orientation and race, ethnicity and racism.

Reporting on sex and gender	N/A
Reporting on race, ethnicity, or other socially relevant groupings	N/A
Population characteristics	N/A
Recruitment	N/A
Ethics oversight	N/A

Note that full information on the approval of the study protocol must also be provided in the manuscript.

Field-specific reporting

Please select the one below that is the best fit for your research. If you are not sure, read the appropriate sections before making your selection.

☒ Life sciences ☐ Behavioural & social sciences ☐ Ecological, evolutionary & environmental sciences

For a reference copy of the document with all sections, see nature.com/documents/nr-reporting-summary-flat.pdf

Life sciences study design

All studies must disclose on these points even when the disclosure is negative.

Sample size	Sample sizes for pyrene-actin polymerization assays and cosedimentation experiments of N = 3 was chosen based on previous experiences with this specific type of experiment and commonly used sample size in the field (Madasu et al 2015, Drazic et al 2018, Zimmet et al 2020, and Carman et al 2022).
Data exclusions	All data corresponding to a technically sound experiments (which did not fail for obvious reasons) are shown. No data were excluded from analysis.
Replication	All biochemical experiments, and western blots, in this study have been reproduced at least three times (in different days) using the sample experimental set-up (proteins and buffers) with similar successful results. For Western blots, all blots are show in the extended data. The data included in the paper represents the last successful replication (N=3) of the experimentation.
Randomization	Not relevant to this work. All experiments were performed with purified protein that was obtained from more than one preparation. Proteins were flash frozen and thawed on the day of the experiment in question.
Blinding	Blinding was not relevant to our study. Since there were numerous conditions to test in biochemical analysis, proper labeling of samples needed to be kept and thus blinding could not be done. For cryo-EM data processing, the initial map that was used for subsequent processing was obtained without a template, a non-bias process that is similar to "blinding" the program. Therefore, the initial map obtained is solely a result of the particles that existed in data.

Reporting for specific materials, systems and methods

We require information from authors about some types of materials, experimental systems and methods used in many studies. Here, indicate whether each material, system or method listed is relevant to your study. If you are not sure if a list item applies to your research, read the appropriate section before selecting a response.

Materials & experimental systems

n/a	Involved in the study
☐	☒ Antibodies
☐	☒ Eukaryotic cell lines
☒	☐ Palaeontology and archaeology
☒	☐ Animals and other organisms
☒	☐ Clinical data
☒	☐ Dual use research of concern
☒	☐ Plants

Methods

n/a	Involved in the study
☒	☐ ChIP-seq
☒	☐ Flow cytometry
☒	☐ MRI-based neuroimaging

Antibodies

Antibodies used

Monoclonal Arp3 antibody was commercially purchased through Santa Cruz Biotechnology (sc-48344) and used in a 1:5000 dilution in this work. Anti-mouse IgG, HRP-linked Antibody was purchased through Cell Signaling Technologies (#7076) and used in a 1:10,000 dilution in this work.

Validation

Arp3 antibody (sc-48344) was validated through HeLa whole cell lysate, A-431 whole cell lysate, and NIH/3T3 whole cell lysate (https://www.scbt.com/p/arp3-antibody-a-1?gclid=CjwKCAjwzJmlBhBBEiwAEJyLu_7_y79gTKt7gqmd_alkXQVmEHinfjqL-uX1t7sF1t3vHKEFVh_v2BoCsaoQAvD_BwE). Anti-mouse IgG HRP-linked Antibody was validated and certification can be found here (https://media.cellsignal.com/coa/7076/38/7076-lot-38-coa.pdf)

Eukaryotic cell lines

Policy information about cell lines and Sex and Gender in Research

Cell line source(s)

Sf9 (ATCC CRL-1711)

Sf9 is a cell line exhibiting epithelial morphology that was derived from pupa ovarian tissue of a fall armyworm. This cell line can be used to replicate baculovirus expression vectors.
Organism: Spodoptera frugiperda, fall armyworm
Tissue: Ovary
Age: pupa
Gender: Female
Morphology: epithelial
Growth properties: Mixed: adherent and suspension

Authentication

Each manufactured batch sf9 cells is authenticated via STR analysis.

Mycoplasma contamination

Mycoplasma contamination was excluded through PCR-based and luminescence-based mycoplasma assays.

Commonly misidentified lines
(See ICLAC register)

Name any commonly misidentified cell lines used in the study and provide a rationale for their use.
